# Paraneoplastic Necrotizing Myelopathy in a Patient With Newly Diagnosed Diffuse Large B Cell Lymphoma

**DOI:** 10.4021/wjon279w

**Published:** 2011-08-24

**Authors:** Chau Hung Lee, Lavina Bharwani, Troy Sullivan

**Affiliations:** a Department of Radiology, Tan Tock Seng Hospital,11 Jalan Tan Tock Seng, Singapore 308433; b John Hopkins Singapore International Medical Centre,11 Jalan Tan Tock Seng, Singapore 308433

**Keywords:** Paraneoplastic myelopathy, Diffuse large B-cell lymphoma

## Abstract

Paraneoplastic neurological disorders are rare but particularly devastating forms of paraneoplastic syndromes, in part due to the fact that the entity is not usually considered as the initial differentials of a cancer patient presenting with neurological symptoms. We report a case of paraneoplastic necrotizing myelopathy associated with Diffuse Large B-cell Lymphoma in an elderly Chinese lady. After extensive investigations, the diagnosis was confirmed on spinal cord biopsy which showed extensive necrotic tissue and absence of tumour or vascular involvement.

## Introduction

A 76-year-old Chinese lady was initially seen at the surgical department for non-specific abdominal pain and anorexia. She had no significant past medial history apart from mild hypertension and hyperlipidemia. A CT scan of the abdomen and pelvis revealed a mass in uncinate process of pancreas and a large soft tissue mass in the pelvis involving the left iliac crest. Biopsy of the left iliac mass showed diffuse large B-cell lymphoma (DLBCL). PET-CT confirmed malignant activity in the left ilium and pancreatic head and showed involvement of several aorto-caval and mesenteric lymph nodes as well. No supradiaphragmatic involvement was detected.

Three-weekly chemotherapy with rituximab, cyclophosphomide, doxorubicin, vincristine, and prednisolone (R-CHOP) was planned. However before treatment could commence, the patient presented with bilateral lower limb weakness and paresthesia rapidly progressing over a few days. She also reported loss of bowel and bladder control. By the time she presented to the emergency department, neurological examination revealed symmetrically dense paraplegia and areflexia in both lower limbs, saddle anesthesia with lax anal tone. Sensory level was noted at around T10. There was no neurological deficit in the upper limbs.

**Figure 1:**
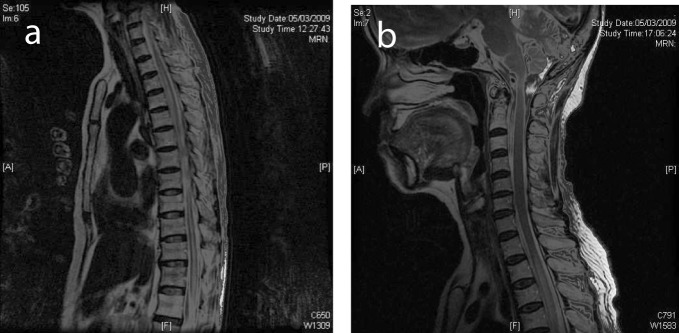
(a) Selected sagittal MR image of the thoracic spine on initial presentation showed normal cord signal. (b) Cervical cord on presentation showed normal MR signal.

Differential diagnoses considered included lymphomatous infiltration of the spinal cord, leptomeningeal spread, cord compression from pathological vertebral fracture, and possibly spinal cord or meningeal infection. Intracranial metastases were also considered. Initial investigations did not indicate any specific etiology. Hemoglobin was 10.7 g/dL. Mild leucocytosis was noted. Serum calcium was slightly raised at 2.7 mmol/L. Hyponatremia was noted with serum sodium of 126 mmol/L. Renal, liver and thyroid function was normal. Serum lactate dehydrogenase was elevated above 3000 U/L likely related to tumour bulk. Chest X-ray was normal. Bone marrow biopsy did not reveal lymphomatous infiltrates. More notable, MRI of the brain and whole spine did not show any acute intracranial or spinal cord abnormality (Fig. 1). Cerebrospinal fluid (CSF) analysis showed cell count, protein and glucose levels within normal limits. No malignant cells were detected. Nevertheless our patient was given a course of intravenous dexamethasone for a presumptive malignant cord compression. Repeat CSF analysis three days later was again normal. Decision was made to proceed with first cycle of chemotherapy (R-CHOP), four days after presentation.

**Figure 2:**
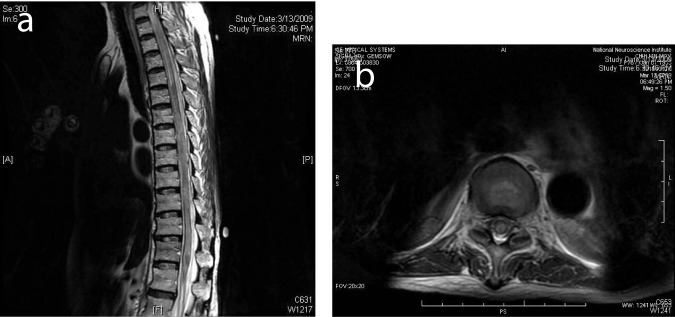
(a) MRI of the spine 1 week after presentation showed diffuse high T2 signal from the spinal cord from T6 to the conus compatible with cord edema. (b) Selected corresponding axial MR image showed edema across the whole cross-section of the spinal cord.

With no neurological improvement, a repeat MRI of the spine obtained a week later showed a long segment of T2-weighted hyperintensity and swelling of the spinal cord with abnormal enhancement from T6 level down to the conus medullaris (Fig. 2). Appearance was compatible with myelitis. Work-up for infective and autoimmune causes was initiated. Blood, CSF microscopy and serology were negative for cytomegalovirus, herpes simplex virus, Ebstein-Barr virus, fungi, and acid-fast bacilli. CSF viral and bacterial cultures were negative. Autoimmune markers, including the neuromyelitis optica (NMO) antibodies, were negative. CSF was again negative for malignant cells. Spinal cord infarct was thought to be less likely due to the long-segment contiguous involvement and lack of significant atherosclerotic disease elsewhere. Decision was made to continue observation as there was no worsening of her neurological deficit clinically.

**Figure 3:**
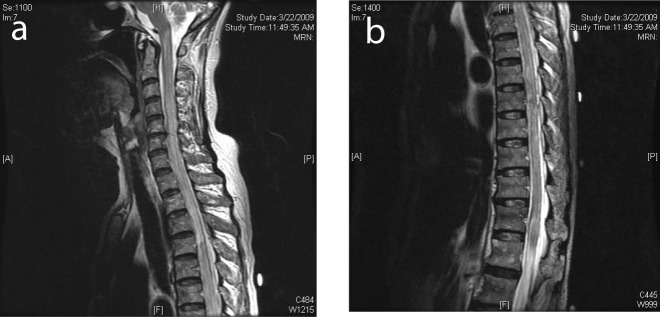
(a) MRI of the spine showing cord edema progressing cranially to involve the cervical spine. (b) MRI demonstrated edema involving the conus medullaris.

Ten days later, the patient complained of bilateral upper limb weakness and paresthesia. Sensory level had progressed to about T7. MRI of the spine now showed interval worsening of abnormal spinal cord enhancement and swelling to T4 level to the conus medullaris and new involvement of the cervical cord (Fig. 3). As no definite explanation could be given for the progression of spinal cord abnormality, a cord biopsy was performed. This showed extensive spinal cord necrosis with no tumour involvement. There was no evidence of thrombosis or inflammation of the spinal vessels. CSF obtained intraoperatively was again negative for infection and malignancy.

After extensive investigations, we concluded that the neurological deficits in our patient and clinical progression were compatible with paraneoplastic necrotizing myelopathy (PNM), as confirmed on spinal cord biopsy.

**Figure 4:**
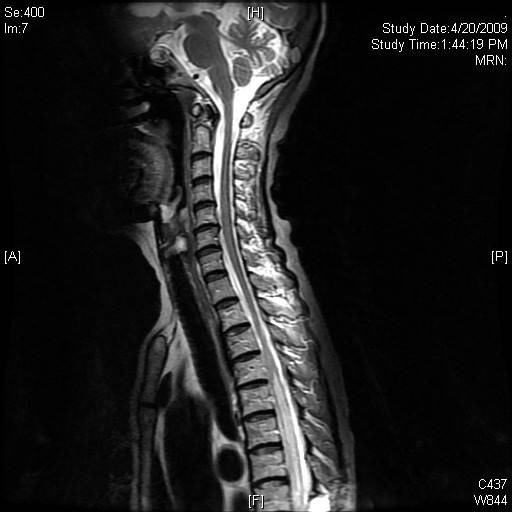
Selected sagittal MR image of the cervical and upper thoracic spine after treatment with methyprednisolone and resumption of chemotherapy, showed near resolution of the abnormal cord signal.

Our patient was started on high dose methyl-prednisolone for five days, on consultation with neurology. She resumed chemotherapy, completing a total of five cycles. MRI performed after the second cycle showed reduction in extent of abnormal spinal cord signal (Fig. 4). Neurological symptoms in the upper limbs gradually resolved. She remained paraplegic with a sensory level at T10. These neurological deficits were stable over the following few months while the visceral disease continued to show response to treatment. Unfortunately she developed severely infected decubitus ulcers and eventually succumbed to sepsis.

## Discussion

PNM is characterised by an ascending, transverse cord dysfunction affecting both anterior and posterior horns of the spinal cord with bilateral motor, sensory and sphincter deficits. It has been hypothesized that the underlying tumor and portions of the nervous system share antigens that generate an autoimmune response against that part of the nervous system resulting in the neurological disorder. Viral infections such as herpes simplex have been implicated in the autoimmune pathogenesis [1, 2]. It has also been observed that malignant lymphomas, particularly Hodgkin lymphoma, are the most frequent malignancies associated with PNM [3].

Clinical presentation of the disorder is mainly of 2 types: Massive cord necrosis presenting as an ascending transverse lesion (usually thoracic), and patchy multifocal necrosis affecting mainly the white matter of the spinal cord similar to that of multiple sclerosis. There is one reported case presenting as a syrinx-like lesion in the upper thoracic cord [3].

Diagnosis is made by exclusion of other known causes of myelopathy. Unlike other paraneoplastic neurologic syndromes there are no associated antibodies or tumour markers to support the diagnosis of PNM. Important differentials to exclude would be that of cord compression from vertebral metastases, infarct, infection, intramedullary or leptomeningeal carcinomatosis, polyradiculopathy, and radiation/chemotherapy-induced damage if specific treatment has been instituted. Demyelinating conditions such as multiple sclerosis or neuromyelitis optica (Devic's disease) should also be considered [4]. MRI reveals signal abnormalities characteristic of cord edema with high T2, low T1 signal, typically affecting the thoracic cord in an ascending fashion.

In the literature, the first case was reviewed by Mancall and Rosales in 1964 [5]. They described two cases of known carcinoma of the lung with acute destruction and degeneration of the spinal cord confirmed on post-mortem. They also analysed eleven patients with various visceral carcinomas presenting with myelopathy, and found similar patterns of progression with ascending severe combined sensory and motor deficits. Of significance is that post-mortem in all the cases did not reveal tumor involvement in any part of the central nervous system, and no changes in the meninges or meningeal and cord vessels. In 1984 Ojeda reported spinal cord necrosis associated with carcinoma of the breast and lung, attributed to paraneoplastic necrotizing myelopathy; no local or systemic causes were found on necropsy [6]. The report also noted just 22 such cases in the English literature at that time. Since then, there have been reports of necropsy-proven PNM associated with a variety of malignancies such as renal cell carcinoma [7], childhood leukemia [8], and multiple myeloma [9]. In Canada there was one report of a patient initially presenting with neurological symptoms suggestive of PNM, where investigations revealed an underlying non-Hodgkin lymphoma [10].

The majority of patients with PNM eventually succumb to respiratory failure from involvement of the cervical cord, the diagnosis of PNM is usually made at autopsy. Treatment of the underlying neoplasm usually fails to halt progression of symptoms. Cases have reported partial response to high dose steroid therapy, intravenous (IV) immunoglobulins, or plasmapheresis [11], but overall treatment response is poor. Hughes reported a case of PNM associated with early Hodgkin's lymphoma that responded well to intrathecal dexamethasone. However he also noted that out of nine other recorded cases of PNM associated with Hodgkin disease, seven failed to respond to various treatment with unremitting progression and eventually death [12]. There is also one report of PNM associated with Hodgkin lymphoma that resolved both clinically and radiologically following successful treatment of the lymphoma [13], but it is believed this is exception rather than the normal.

There are few reported cases of PNM the Asia-Pacific region. The Japanese have published a case of necrotizing myelopathy associated with hepatocellular carcinoma [14], as well as with esophageal cancer [15]. In Australia there have been isolated reports of PNM associated with Hodgkin lymphoma. Our patient is probably the first biopsy-proven case of PNM in a living subject, associated with non-Hodgkin lymphoma. Unlike most cases of PNM in the literature which usually runs a progressive course, our patient remained neurologically stable with high-dose steroids and control of the malignant disease.

### Conclusion

Primary MRI abnormality in PNM is that of spinal cord edema. This appearance is non-specific and can be due to a variety of causes such as spinal cord infarct, malignant infiltration, infective myelitis or demyelinating disorders. However, in the appropriate clinical context, it is important to consider the possibility of PNM, even if initial MRI studies do not reveal any abnormality. PNM should also be considered in previously well patients with unexplained cord symptoms that could be the initial presentation of an occult malignancy.
